# *tert*-Butyl 5-{2-[2-(*N*-ethynyl-4-methyl­benzene­sulfonamido)­phen­yl]ethyn­yl}furan-2-carboxyl­ate

**DOI:** 10.1107/S2414314625006558

**Published:** 2025-08-15

**Authors:** Benjamin Dassonneville, Heiner Detert, Dieter Schollmeyer

**Affiliations:** a University Mainz, Duesbergweg 10-14, 55099 Mainz, Germany; University of Aberdeen, United Kingdom

**Keywords:** crystal structure, ynamide, diyne, furane

## Abstract

The crystal structure of an *o*,*N*-dialkynyl­tosyl­aniline, C_26_H_23_NO_5_S, is presented. Two essentially planar and nearly parallel branches are connected to the aniline unit and the angle between the alkynes amounts to 26 (4)°. Weak intra­molecular aromatic π–π stacking occurs.

## Abstract 2

In the title mol­ecule, two essentially planar and nearly parallel branches are connected to the aniline unit and the angle between the alkynes amounts to 26 (4)°. Weak intra­molecular aromatic π–π stacking occurs.

## Graphical abstract

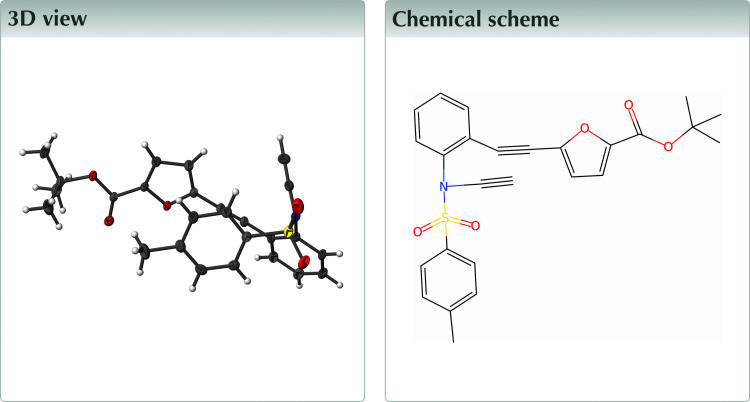

## Structure description

In a project on heterocarbazoles (Letessier *et al.*, 2012 ▸, 2013 ▸; Letessier & Detert, 2012 ▸), the title compound, C_26_H_23_NO_5_S (**I**), was prepared as substrate for a study on rhodium-catalyzed 2 + 2 + 2 cyclo­additions of alkynylynamides to carbolines (Nissen & Detert, 2011 ▸) and indolo­thio­pyranes (Dassonneville *et al.*, 2023*a* ▸,*b* ▸). This compound is a key inter­mediate in the synthesis of isoperlolyrine (Dassonneville *et al.*, 2011 ▸).

**Figure 1:**
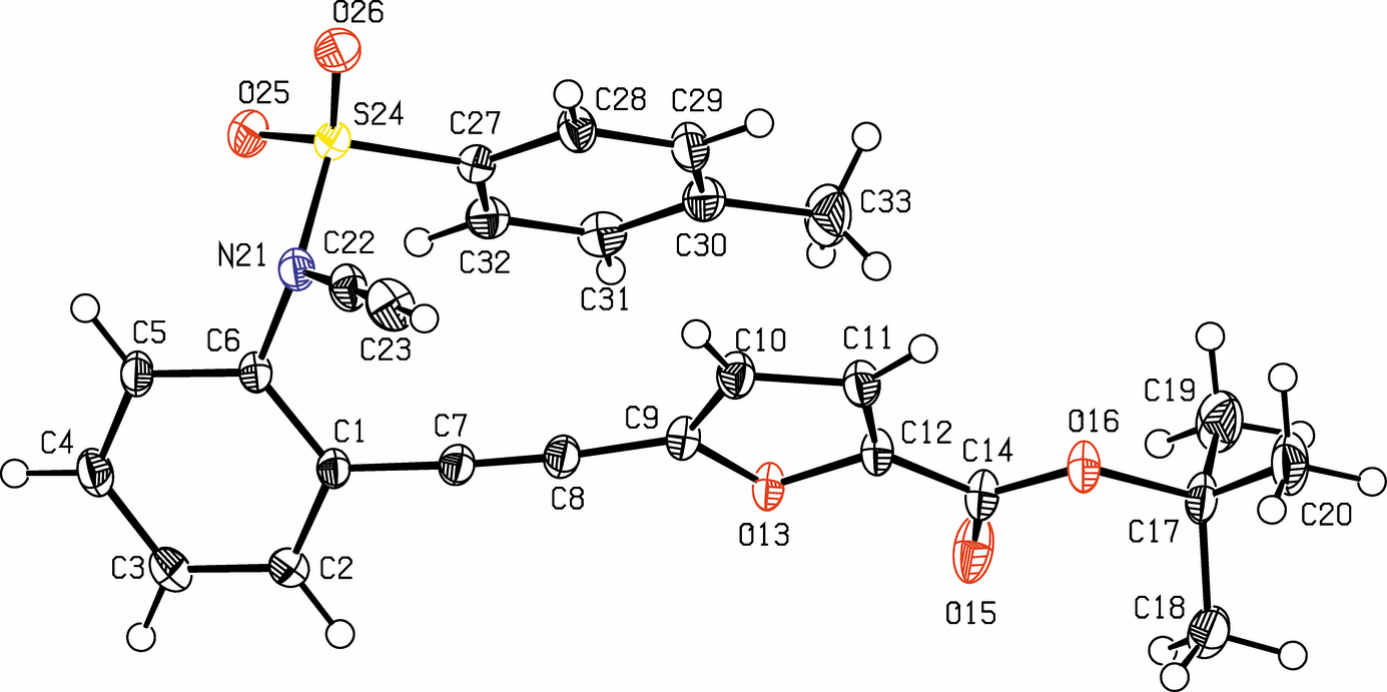
The mol­ecular structure of (**I**) with displacement ellipsoids drawn at the 50% probability level.

**Figure 2:**
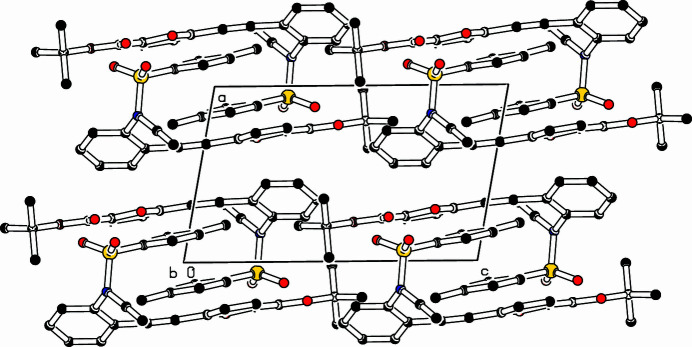
Part of the packing diagram viewed along the *b*-axis direction. Hydrogen atoms omitted for clarity.

The crystal structure of (**I**) shows (Fig. 1 ▸) that two near planar segments are connected to the *N*-ethynylaniline unit. The furan ring (C9–C12/O13) and the tolyl ring (C27–C32) include an angle of 9.01 (5)°; the distance between the centroids of these rings is 4.0830 (5) Å with a slippage of 2.105 Å. The alkynyl­furan­carb­oxy­lic ester unit (C7–C12/O13/C14/O15) is essentially planar with a maximum deviation of 0.1111 (8) Å at C1. The dihedral angle between the furan (C9–C12/O13) and aniline ring (C1–C6) is 33.53 (5) ° and the alkyne units N21/C22/C23 and C1/C7/C8/C9 subtend an angle of 26 (4)°. In the crystal, two mol­ecules fill the triclinic unit cell and neighboring mol­ecules are connected *via* van der Waals inter­actions and a center of inversion (Fig. 2 ▸).

## Synthesis and crystallization

Synthetic details are given in the literature (Dassonneville *et al.*, 2011 ▸). Assignment of signals is based on two-dimensional NMR, notation follows IUPAC nomenclature. The compound was obtained as white solid, m.p.= 384–385 K. ^1^H NMR (400 MHz, CDCl_3_, 298 K):7.69 (*d*, ^3^*J*_H,H_ = 8.2 Hz, 2H, 2-H, 6-H Ts), 7.49 (*d*, ^3^*J*_H,H_ = 7.0 Hz, 1H, 3-H), 7.43 (*m*, 3H, 4-H, 5-H, 6-H), 7.21 (*d*, ^3^*J*_H,H_ = 8.2 Hz, 2H, 3-H, 5-H Ts), 7.04 (*d*, ^3^*J*_H,H_ = 3.5 Hz, 1H, 4-H Fu), 6.53 (*d*, ^3^*J*_H,H_ = 3.5 Hz, 1H, 3-H Fu), 2.90 (*s*, 1H, 8-H), 2.31 (*s*, 3H, CH_3_ Ts), 1.60 (*s*, 9H, ^*t*^Bu). ^13^C NMR (CDCl3): 157.2 (C_q_, COOtBu), 145.9 (C_q_, C-5 Fu), 145.1 (C_q_, C-4 T s), 139.2 (C_q_, C-1), 138.3 (C_q_, C-2 Fu), 133.9 (C_q_, C-1 T s), 133.3 (CH, C-3), 130.1 (CH, C-4), 130.0 (CH, C-6), 129.7 (CH, C-3, C-5 T s), 129.2 (CH, C-5), 128.3 (CH, C-2, C-6 T s), 121.2 (C_q_, C-2), 117.7 (CH, C-4 Fu), 117.2 (CH, C-3 Fu), 90.0 (C_q_ Ph—CC—Fu), 84.4 (C_q_, Ph—CC—Fu), 82.3 (C_q_, C(CH_3_)3), 75.4 (C_q_, N—CC—H), 59.3 (CH, N—CC—H), 28.2 (CH_3_, tBu), 21.5 (CH_3_ Ts). IR (neat): 3289, 2988, 2129, 1722, 1516, 1375, 1307, 1173, 1137, 1010, 766 cm^−1^. FD–MS: *m*/*z* (%) = 461.0 (100) [*M*]^+.^ C_26_H_23_NO_5_S (461.13) calculated C 67.66, H 5.02, N 3.03, S 6.95; found C 67.43, H 4.93, N 3.01, S 6.67.

## Refinement

Crystal data, data collection and structure refinement details are summarized in Table 1 ▸.

## Supplementary Material

Crystal structure: contains datablock(s) I, global. DOI: 10.1107/S2414314625006558/hb4528sup1.cif

Structure factors: contains datablock(s) I. DOI: 10.1107/S2414314625006558/hb4528Isup2.hkl

Supporting information file. DOI: 10.1107/S2414314625006558/hb4528Isup3.cml

CCDC reference: 2474369

Additional supporting information:  crystallographic information; 3D view; checkCIF report

## Figures and Tables

**Table 1:** Experimental details

Crystal data	
Chemical formula	C_26_H_23_NO_5_S
*M*_r_	461.51
Crystal system, space group	Triclinic, *P* 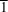
Temperature (K)	120
*a*, *b*, *c* (Å)	7.7527 (3), 12.6007 (5), 13.7529 (5)
α, β, γ (°)	66.199 (3), 76.607 (3), 80.350 (3)
*V* (Å^3^)	1191.66 (8)
*Z*	2
Radiation type	Mo *K*α
μ (mm^−1^)	0.17
Crystal size (mm)	0.51 × 0.45 × 0.37

Data collection	
Diffractometer	Stoe Stadivsri
Absorption correction	Integration
*T*_min_, *T*_max_	0.899, 0.955
No. of measured, independent and observed [*I* > 2σ(*I*)] reflections	21571, 8903, 7763
*R*_int_	0.018
(sin θ/λ)_max_ (Å^−1^)	0.804

Refinement	
*R*[*F*^2^ > 2σ(*F*^2^)], *wR*(*F*^2^), *S*	0.037, 0.114, 1.06
No. of reflections	8903
No. of parameters	302
H-atom treatment	H-atom parameters constrained
Δρ_max_, Δρ_min_ (e Å^−3^)	0.43, −0.50

Computer programs: *X-AREAWinXpose*, *Recipe* and *Integrate* (Stoe & Cie, 2020 ▸), *SHELXT2014* (Sheldrick, 2015*a* ▸), *SHELXL2019/2* (Sheldrick, 2015*b* ▸) and *PLATON* (Spek, 2009 ▸).

## full crystallographic data

### tert-Butyl 5-{2-[2-(N-ethynyl-4-methylbenzenesulfonamido)phenyl]ethynyl}furan-2-carboxylate Crystal data

**Table:**

C_26_H_23_NO_5_S	*F*(000) = 484
*M**_r_* = 461.51	*D*_x_ = 1.286 Mg m^−^^3^
Triclinic, *P*1	Melting point: 384 K
*a* = 7.7527 (3) Å	Mo *K*α radiation, λ = 0.71073 Å
*b* = 12.6007 (5) Å	Cell parameters from 33755 reflections
*c* = 13.7529 (5) Å	θ = 2.7–34.8°
α = 66.199 (3)°	µ = 0.17 mm^−^^1^
β = 76.607 (3)°	*T* = 120 K
γ = 80.350 (3)°	Block, colorless
*V* = 1191.66 (8) Å^3^	0.51 × 0.45 × 0.37 mm
*Z* = 2	

### tert-Butyl 5-{2-[2-(N-ethynyl-4-methylbenzenesulfonamido)phenyl]ethynyl}furan-2-carboxylate Data collection

**Table:**

Stoe Stadivsri diffractometer	8903 independent reflections
Radiation source: Axo Mo	7763 reflections with *I* > 2σ(*I*)
Detector resolution: 13.33 pixels mm^-1^	*R*_int_ = 0.018
rotation method, ω scans	θ_max_ = 34.8°, θ_min_ = 2.7°
Absorption correction: integration	*h* = −12→12
*T*_min_ = 0.899, *T*_max_ = 0.955	*k* = −18→18
21571 measured reflections	*l* = −19→21

### tert-Butyl 5-{2-[2-(N-ethynyl-4-methylbenzenesulfonamido)phenyl]ethynyl}furan-2-carboxylate Refinement

**Table:**

Refinement on *F*^2^	Primary atom site location: dual
Least-squares matrix: full	Hydrogen site location: inferred from neighbouring sites
*R*[*F*^2^ > 2σ(*F*^2^)] = 0.037	H-atom parameters constrained
*wR*(*F*^2^) = 0.114	*w* = 1/[σ^2^(*F*_o_^2^) + (0.0733*P*)^2^ + 0.1217*P*] where *P* = (*F*_o_^2^ + 2*F*_c_^2^)/3
*S* = 1.06	(Δ/σ)_max_ = 0.001
8903 reflections	Δρ_max_ = 0.43 e Å^−^^3^
302 parameters	Δρ_min_ = −0.50 e Å^−^^3^
0 restraints	

### tert-Butyl 5-{2-[2-(N-ethynyl-4-methylbenzenesulfonamido)phenyl]ethynyl}furan-2-carboxylate Special details

**Table:**

Geometry. All esds (except the esd in the dihedral angle between two l.s. planes) are estimated using the full covariance matrix. The cell esds are taken into account individually in the estimation of esds in distances, angles and torsion angles; correlations between esds in cell parameters are only used when they are defined by crystal symmetry. An approximate (isotropic) treatment of cell esds is used for estimating esds involving l.s. planes.
Refinement. Hydrogen atoms attached to carbon atoms were placed at calculated positions and were refined in the riding-model approximation with C—H = 0.99 Å, and with *U*_iso_(H) = 1.2 *U*_eq_(C).

### tert-Butyl 5-{2-[2-(N-ethynyl-4-methylbenzenesulfonamido)phenyl]ethynyl}furan-2-carboxylate Fractional atomic coordinates and isotropic or equivalent isotropic displacement parameters (Å^2^)

**Table:**

	*x*	*y*	*z*	*U*_iso_*/*U*_eq_	
C1	0.65107 (10)	0.32278 (7)	−0.19892 (6)	0.01866 (13)	
C2	0.55638 (11)	0.26355 (7)	−0.23392 (7)	0.02295 (14)	
H2	0.492155	0.200048	−0.182434	0.028*	
C3	0.55562 (12)	0.29685 (8)	−0.34311 (7)	0.02667 (16)	
H3	0.490861	0.256310	−0.366082	0.032*	
C4	0.64960 (12)	0.38951 (8)	−0.41885 (7)	0.02534 (15)	
H4	0.650538	0.411245	−0.493626	0.030*	
C5	0.74236 (11)	0.45066 (7)	−0.38581 (6)	0.02202 (14)	
H5	0.805253	0.514593	−0.437702	0.026*	
C6	0.74237 (10)	0.41758 (6)	−0.27644 (6)	0.01835 (13)	
C7	0.65771 (10)	0.28461 (7)	−0.08693 (6)	0.02025 (14)	
C8	0.67369 (11)	0.25010 (7)	0.00618 (6)	0.02139 (14)	
C9	0.70200 (11)	0.21554 (7)	0.11283 (6)	0.02034 (13)	
C10	0.73297 (12)	0.27686 (7)	0.16843 (6)	0.02385 (15)	
H10	0.735045	0.358862	0.143447	0.029*	
C11	0.76142 (11)	0.19355 (7)	0.27098 (6)	0.02309 (15)	
H11	0.787304	0.208616	0.328216	0.028*	
C12	0.74427 (11)	0.08751 (7)	0.27112 (6)	0.02079 (14)	
O13	0.70783 (8)	0.09883 (5)	0.17451 (4)	0.02131 (11)	
C14	0.75674 (12)	−0.03284 (7)	0.35009 (6)	0.02299 (15)	
O15	0.74907 (12)	−0.11598 (6)	0.32893 (5)	0.03557 (17)	
O16	0.77637 (9)	−0.03494 (5)	0.44483 (5)	0.02390 (12)	
C17	0.79837 (13)	−0.14879 (7)	0.53537 (6)	0.02594 (16)	
C18	0.64352 (15)	−0.22090 (9)	0.56027 (7)	0.03345 (19)	
H18A	0.532091	−0.170721	0.557720	0.050*	
H18B	0.639044	−0.282360	0.632535	0.050*	
H18C	0.659550	−0.256150	0.506632	0.050*	
C19	0.97710 (15)	−0.21039 (10)	0.50736 (9)	0.0397 (2)	
H19A	0.976867	−0.230521	0.445612	0.060*	
H19B	0.998354	−0.281528	0.569635	0.060*	
H19C	1.071458	−0.158875	0.488766	0.060*	
C20	0.79498 (18)	−0.11173 (10)	0.62842 (8)	0.0389 (2)	
H20A	0.893700	−0.063671	0.610406	0.058*	
H20B	0.807097	−0.180978	0.694009	0.058*	
H20C	0.681962	−0.066703	0.640712	0.058*	
N21	0.83817 (9)	0.48321 (6)	−0.24387 (5)	0.02043 (12)	
C22	0.75138 (12)	0.52801 (7)	−0.16914 (7)	0.02340 (15)	
C23	0.67232 (13)	0.56911 (8)	−0.10549 (8)	0.02971 (17)	
H23	0.609321	0.601863	−0.054762	0.036*	
S24	1.06219 (3)	0.45912 (2)	−0.25700 (2)	0.02193 (6)	
O25	1.11953 (9)	0.43671 (7)	−0.35390 (5)	0.02992 (14)	
O26	1.11886 (10)	0.55567 (6)	−0.24649 (6)	0.03253 (15)	
C27	1.10259 (10)	0.33213 (7)	−0.14669 (6)	0.01959 (13)	
C28	1.12413 (12)	0.33950 (7)	−0.05187 (7)	0.02338 (15)	
H28	1.115412	0.412941	−0.046160	0.028*	
C29	1.15851 (12)	0.23764 (8)	0.03392 (7)	0.02635 (16)	
H29	1.174212	0.241690	0.098796	0.032*	
C30	1.17050 (11)	0.12906 (7)	0.02674 (7)	0.02526 (15)	
C31	1.14652 (12)	0.12424 (7)	−0.06865 (7)	0.02553 (16)	
H31	1.153974	0.050772	−0.074146	0.031*	
C32	1.11193 (11)	0.22493 (7)	−0.15577 (7)	0.02261 (14)	
H32	1.094929	0.220920	−0.220394	0.027*	
C33	1.21211 (17)	0.01939 (9)	0.12007 (9)	0.0400 (2)	
H33A	1.214581	−0.048230	0.101444	0.060*	
H33B	1.120403	0.013194	0.184114	0.060*	
H33C	1.328395	0.021903	0.135330	0.060*	

### tert-Butyl 5-{2-[2-(N-ethynyl-4-methylbenzenesulfonamido)phenyl]ethynyl}furan-2-carboxylate Atomic displacement parameters (Å^2^)

**Table:**

	*U*^11^	*U*^22^	*U*^33^	*U*^12^	*U*^13^	*U*^23^
C1	0.0189 (3)	0.0192 (3)	0.0153 (3)	−0.0005 (2)	−0.0048 (2)	−0.0035 (2)
C2	0.0215 (3)	0.0238 (3)	0.0229 (3)	−0.0040 (3)	−0.0069 (3)	−0.0058 (3)
C3	0.0273 (4)	0.0314 (4)	0.0254 (4)	−0.0037 (3)	−0.0107 (3)	−0.0112 (3)
C4	0.0289 (4)	0.0305 (4)	0.0181 (3)	−0.0009 (3)	−0.0091 (3)	−0.0087 (3)
C5	0.0266 (3)	0.0227 (3)	0.0144 (3)	−0.0018 (3)	−0.0060 (3)	−0.0036 (3)
C6	0.0209 (3)	0.0183 (3)	0.0150 (3)	−0.0006 (2)	−0.0057 (2)	−0.0046 (2)
C7	0.0207 (3)	0.0197 (3)	0.0173 (3)	−0.0014 (2)	−0.0038 (2)	−0.0038 (3)
C8	0.0230 (3)	0.0207 (3)	0.0172 (3)	−0.0013 (3)	−0.0039 (2)	−0.0039 (3)
C9	0.0242 (3)	0.0189 (3)	0.0154 (3)	−0.0010 (2)	−0.0046 (2)	−0.0037 (2)
C10	0.0316 (4)	0.0187 (3)	0.0205 (3)	−0.0027 (3)	−0.0079 (3)	−0.0048 (3)
C11	0.0295 (4)	0.0222 (3)	0.0186 (3)	−0.0017 (3)	−0.0079 (3)	−0.0070 (3)
C12	0.0277 (4)	0.0197 (3)	0.0138 (3)	0.0003 (3)	−0.0060 (2)	−0.0050 (2)
O13	0.0311 (3)	0.0183 (2)	0.0133 (2)	−0.0011 (2)	−0.0060 (2)	−0.00406 (19)
C14	0.0324 (4)	0.0202 (3)	0.0144 (3)	0.0005 (3)	−0.0053 (3)	−0.0049 (3)
O15	0.0664 (5)	0.0211 (3)	0.0198 (3)	−0.0015 (3)	−0.0098 (3)	−0.0080 (2)
O16	0.0361 (3)	0.0198 (3)	0.0150 (2)	−0.0003 (2)	−0.0090 (2)	−0.0043 (2)
C17	0.0354 (4)	0.0220 (4)	0.0158 (3)	−0.0018 (3)	−0.0086 (3)	−0.0006 (3)
C18	0.0424 (5)	0.0314 (4)	0.0222 (4)	−0.0104 (4)	−0.0058 (3)	−0.0028 (3)
C19	0.0388 (5)	0.0319 (5)	0.0348 (5)	0.0071 (4)	−0.0106 (4)	−0.0005 (4)
C20	0.0603 (7)	0.0381 (5)	0.0194 (4)	−0.0089 (5)	−0.0157 (4)	−0.0055 (4)
N21	0.0248 (3)	0.0193 (3)	0.0175 (3)	−0.0024 (2)	−0.0063 (2)	−0.0058 (2)
C22	0.0302 (4)	0.0184 (3)	0.0218 (3)	−0.0002 (3)	−0.0102 (3)	−0.0057 (3)
C23	0.0339 (4)	0.0285 (4)	0.0319 (4)	0.0017 (3)	−0.0097 (3)	−0.0164 (4)
S24	0.02414 (10)	0.02249 (10)	0.01708 (9)	−0.00750 (7)	−0.00447 (6)	−0.00286 (7)
O25	0.0271 (3)	0.0417 (4)	0.0170 (3)	−0.0068 (3)	−0.0005 (2)	−0.0075 (2)
O26	0.0395 (4)	0.0240 (3)	0.0332 (3)	−0.0138 (3)	−0.0139 (3)	−0.0016 (3)
C27	0.0197 (3)	0.0208 (3)	0.0180 (3)	−0.0032 (2)	−0.0035 (2)	−0.0064 (3)
C28	0.0304 (4)	0.0210 (3)	0.0210 (3)	−0.0020 (3)	−0.0091 (3)	−0.0079 (3)
C29	0.0337 (4)	0.0246 (4)	0.0213 (3)	−0.0021 (3)	−0.0108 (3)	−0.0063 (3)
C30	0.0245 (4)	0.0215 (3)	0.0262 (4)	−0.0010 (3)	−0.0065 (3)	−0.0048 (3)
C31	0.0259 (4)	0.0209 (3)	0.0303 (4)	−0.0010 (3)	−0.0038 (3)	−0.0113 (3)
C32	0.0229 (3)	0.0246 (4)	0.0224 (3)	−0.0028 (3)	−0.0028 (3)	−0.0115 (3)
C33	0.0497 (6)	0.0256 (4)	0.0370 (5)	0.0020 (4)	−0.0172 (4)	−0.0011 (4)

### tert-Butyl 5-{2-[2-(N-ethynyl-4-methylbenzenesulfonamido)phenyl]ethynyl}furan-2-carboxylate Geometric parameters (Å, º)

**Table:**

C1—C2	1.4018 (11)	C18—H18B	0.9800
C1—C6	1.4037 (10)	C18—H18C	0.9800
C1—C7	1.4272 (10)	C19—H19A	0.9800
C2—C3	1.3878 (11)	C19—H19B	0.9800
C2—H2	0.9500	C19—H19C	0.9800
C3—C4	1.3896 (13)	C20—H20A	0.9800
C3—H3	0.9500	C20—H20B	0.9800
C4—C5	1.3918 (11)	C20—H20C	0.9800
C4—H4	0.9500	N21—C22	1.3594 (11)
C5—C6	1.3894 (10)	N21—S24	1.6921 (7)
C5—H5	0.9500	C22—C23	1.1922 (12)
C6—N21	1.4475 (10)	C23—H23	0.9500
C7—C8	1.2043 (11)	S24—O25	1.4283 (7)
C8—C9	1.4109 (10)	S24—O26	1.4299 (7)
C9—C10	1.3651 (11)	S24—C27	1.7472 (8)
C9—O13	1.3685 (9)	C27—C28	1.3931 (10)
C10—C11	1.4182 (11)	C27—C32	1.3946 (11)
C10—H10	0.9500	C28—C29	1.3854 (11)
C11—C12	1.3639 (11)	C28—H28	0.9500
C11—H11	0.9500	C29—C30	1.3974 (12)
C12—O13	1.3685 (9)	C29—H29	0.9500
C12—C14	1.4693 (11)	C30—C31	1.3930 (12)
C14—O15	1.2086 (10)	C30—C33	1.5073 (13)
C14—O16	1.3357 (9)	C31—C32	1.3875 (12)
O16—C17	1.4873 (10)	C31—H31	0.9500
C17—C19	1.5180 (14)	C32—H32	0.9500
C17—C18	1.5193 (14)	C33—H33A	0.9800
C17—C20	1.5228 (13)	C33—H33B	0.9800
C18—H18A	0.9800	C33—H33C	0.9800
			
C2—C1—C6	118.61 (7)	H18B—C18—H18C	109.5
C2—C1—C7	120.18 (7)	C17—C19—H19A	109.5
C6—C1—C7	121.19 (7)	C17—C19—H19B	109.5
C3—C2—C1	120.59 (8)	H19A—C19—H19B	109.5
C3—C2—H2	119.7	C17—C19—H19C	109.5
C1—C2—H2	119.7	H19A—C19—H19C	109.5
C2—C3—C4	119.99 (8)	H19B—C19—H19C	109.5
C2—C3—H3	120.0	C17—C20—H20A	109.5
C4—C3—H3	120.0	C17—C20—H20B	109.5
C3—C4—C5	120.40 (7)	H20A—C20—H20B	109.5
C3—C4—H4	119.8	C17—C20—H20C	109.5
C5—C4—H4	119.8	H20A—C20—H20C	109.5
C6—C5—C4	119.56 (7)	H20B—C20—H20C	109.5
C6—C5—H5	120.2	C22—N21—C6	119.08 (7)
C4—C5—H5	120.2	C22—N21—S24	117.23 (6)
C5—C6—C1	120.84 (7)	C6—N21—S24	119.20 (5)
C5—C6—N21	118.60 (7)	C23—C22—N21	178.24 (9)
C1—C6—N21	120.56 (6)	C22—C23—H23	180.0
C8—C7—C1	176.13 (8)	O25—S24—O26	121.34 (4)
C7—C8—C9	175.78 (9)	O25—S24—N21	104.93 (4)
C10—C9—O13	110.83 (7)	O26—S24—N21	105.16 (4)
C10—C9—C8	132.13 (7)	O25—S24—C27	109.12 (4)
O13—C9—C8	116.98 (7)	O26—S24—C27	109.34 (4)
C9—C10—C11	106.20 (7)	N21—S24—C27	105.74 (4)
C9—C10—H10	126.9	C28—C27—C32	121.37 (7)
C11—C10—H10	126.9	C28—C27—S24	119.82 (6)
C12—C11—C10	106.38 (7)	C32—C27—S24	118.81 (6)
C12—C11—H11	126.8	C29—C28—C27	118.72 (7)
C10—C11—H11	126.8	C29—C28—H28	120.6
C11—C12—O13	110.77 (7)	C27—C28—H28	120.6
C11—C12—C14	134.61 (7)	C28—C29—C30	121.17 (8)
O13—C12—C14	114.62 (7)	C28—C29—H29	119.4
C9—O13—C12	105.83 (6)	C30—C29—H29	119.4
O15—C14—O16	126.62 (7)	C31—C30—C29	118.88 (8)
O15—C14—C12	122.82 (7)	C31—C30—C33	120.74 (8)
O16—C14—C12	110.56 (7)	C29—C30—C33	120.37 (8)
C14—O16—C17	119.45 (6)	C32—C31—C30	121.11 (8)
O16—C17—C19	108.92 (7)	C32—C31—H31	119.4
O16—C17—C18	110.75 (7)	C30—C31—H31	119.4
C19—C17—C18	112.81 (9)	C31—C32—C27	118.75 (7)
O16—C17—C20	102.12 (7)	C31—C32—H32	120.6
C19—C17—C20	111.31 (9)	C27—C32—H32	120.6
C18—C17—C20	110.42 (8)	C30—C33—H33A	109.5
C17—C18—H18A	109.5	C30—C33—H33B	109.5
C17—C18—H18B	109.5	H33A—C33—H33B	109.5
H18A—C18—H18B	109.5	C30—C33—H33C	109.5
C17—C18—H18C	109.5	H33A—C33—H33C	109.5
H18A—C18—H18C	109.5	H33B—C33—H33C	109.5
			
C6—C1—C2—C3	1.09 (12)	C14—O16—C17—C20	−173.94 (8)
C7—C1—C2—C3	−177.15 (8)	C5—C6—N21—C22	126.08 (8)
C1—C2—C3—C4	0.14 (13)	C1—C6—N21—C22	−53.72 (10)
C2—C3—C4—C5	−1.10 (14)	C5—C6—N21—S24	−78.34 (8)
C3—C4—C5—C6	0.80 (13)	C1—C6—N21—S24	101.86 (8)
C4—C5—C6—C1	0.45 (12)	C22—N21—S24—O25	−168.07 (6)
C4—C5—C6—N21	−179.35 (7)	C6—N21—S24—O25	35.91 (7)
C2—C1—C6—C5	−1.38 (11)	C22—N21—S24—O26	−39.02 (7)
C7—C1—C6—C5	176.84 (7)	C6—N21—S24—O26	164.96 (6)
C2—C1—C6—N21	178.42 (7)	C22—N21—S24—C27	76.63 (7)
C7—C1—C6—N21	−3.37 (11)	C6—N21—S24—C27	−79.40 (6)
O13—C9—C10—C11	0.49 (10)	O25—S24—C27—C28	155.33 (7)
C8—C9—C10—C11	−176.59 (9)	O26—S24—C27—C28	20.49 (8)
C9—C10—C11—C12	−0.53 (10)	N21—S24—C27—C28	−92.27 (7)
C10—C11—C12—O13	0.40 (10)	O25—S24—C27—C32	−24.89 (7)
C10—C11—C12—C14	−179.88 (9)	O26—S24—C27—C32	−159.73 (7)
C10—C9—O13—C12	−0.25 (9)	N21—S24—C27—C32	87.51 (7)
C8—C9—O13—C12	177.32 (7)	C32—C27—C28—C29	1.12 (13)
C11—C12—O13—C9	−0.11 (9)	S24—C27—C28—C29	−179.11 (7)
C14—C12—O13—C9	−179.89 (7)	C27—C28—C29—C30	−0.42 (14)
C11—C12—C14—O15	−174.32 (10)	C28—C29—C30—C31	−0.25 (14)
O13—C12—C14—O15	5.40 (13)	C28—C29—C30—C33	178.61 (9)
C11—C12—C14—O16	5.91 (14)	C29—C30—C31—C32	0.25 (13)
O13—C12—C14—O16	−174.38 (7)	C33—C30—C31—C32	−178.62 (9)
O15—C14—O16—C17	2.46 (14)	C30—C31—C32—C27	0.43 (13)
C12—C14—O16—C17	−177.77 (7)	C28—C27—C32—C31	−1.13 (12)
C14—O16—C17—C19	68.25 (10)	S24—C27—C32—C31	179.10 (6)
C14—O16—C17—C18	−56.37 (10)		
